# SIRT1 exerts protective effects by inhibiting endoplasmic reticulum stress and NF-κB signaling pathways

**DOI:** 10.3389/fcell.2024.1405546

**Published:** 2024-04-30

**Authors:** Kaixuan Zhao, Haoyue Zhang, Dong Yang

**Affiliations:** Plastic Surgery Hospital (PSH), Chinese Academy of Medical Sciences and Peking Union Medical College, Beijing, China

**Keywords:** SIRT1, endoplasmic reticulum stress, nuclear factor-κB, deacetylation, metabolic diseases, neurodegenerative diseases

## Abstract

Silent information regulator two homolog 1 (SIRT1), an NAD + -dependent histone deacetylase, plays a pivotal regulatory role in a myriad of physiological processes. A growing body of evidence suggests that SIRT1 can exert protective effects in metabolic disorders and neurodegenerative diseases by inhibiting endoplasmic reticulum (ER) stress and the nuclear factor-κB (NF-κB) inflammatory signaling pathway. This review systematically elucidates the molecular mechanisms and biological significance of SIRT1 in regulating ER stress and the NF-κB pathway. On one hand, SIRT1 can deacetylate key molecules in the ER stress pathway, such as glucose-regulated protein 78 (GRP78), X-box binding protein 1 (XBP1), PKR-like ER kinase (PERK), inositol-requiring enzyme 1α (IRE1α), and activating transcription factor 6 (ATF6), thereby alleviating ER stress. On the other hand, SIRT1 can directly or indirectly remove the acetylation modification of the NF-κB p65 subunit, inhibiting its transcriptional activity and thus attenuating inflammatory responses. Through these mechanisms, SIRT1 can ameliorate insulin resistance in metabolic diseases, exert cardioprotective effects in ischemia-reperfusion injury, and reduce neuronal damage in neurodegenerative diseases. However, it is important to note that while these findings are promising, the complex nature of the biological systems involved warrants further investigation to fully unravel the intricacies of SIRT1’s regulatory mechanisms. Nevertheless, understanding the regulatory mechanisms of SIRT1 on ER stress and the NF-κB pathway is of great significance for expanding our knowledge of the pathogenesis of related diseases and exploring new preventive and therapeutic strategies targeting SIRT1.

## 1 Introduction

SIRT1, a prominent member of the mammalian sirtuins family, is an NAD + -dependent histone deacetylase that widely participates in the epigenetic regulation of various biological processes, such as cellular stress, metabolism, and inflammation (Chalkiadaki and Guarente, 2015).

SIRT1 can remove acetyl groups directly from histones and a number of non-histone substrates, thus modulating gene expression and protein function (Chang and Guarente, 2014). It is worth noting that numerous studies have revealed the critical protective role of SIRT1 in the pathological processes such as metabolic diseases and neurodegenerative diseases which is mainly due to its inhibition of ER stress and the NF-κB inflammatory pathway (Prola et al., 2017). On the other hand, the intricacy of the biological processes involved and the possibility of confounding factors requires a careful interpretation of these results.

The endoplasmic reticulum (ER) is a vital organelle where proteins are synthesized, folded, and matured before they are secreted or inserted into the cell membrane. ER stress is a condition when the ER protein folding capability is overwhelmed causing the accumulation of the unfolded or misfolded proteins in the ER lumen (Hetz and Papa, 2018). This can be caused by various physiological and pathological factors, including nutrient deprivation, oxidative stress, calcium imbalance, and genetic mutations affecting protein folding (Cybulsky, 2017).

To cope with ER stress, cells activate the unfolded protein response (UPR), an adaptive signaling cascade aimed at restoring ER homeostasis. The UPR is mediated by three main sensors located on the ER membrane: PKR-like ER kinase (PERK), inositol-requiring enzyme 1α (IRE1α), and activating transcription factor 6 (ATF6) (Hetz and Papa, 2018). Under normal conditions, these sensors are kept inactive by binding to the ER chaperone glucose-regulated protein 78 (GRP78). However, during ER stress, GRP78 dissociates from the sensors to assist in protein folding, leading to their activation and the initiation of downstream signaling cascades (Cybulsky, 2017).

The UPR aims to alleviate ER stress through several mechanisms, including reducing protein synthesis, enhancing ER folding capacity, and promoting the degradation of misfolded proteins via the ER-associated degradation (ERAD) pathway. However, if ER stress persists and cannot be resolved, the UPR can trigger apoptotic cell death (Hetz and Papa, 2018). Chronic ER stress has been implicated in the pathogenesis of various diseases, such as metabolic disorders, neurodegenerative diseases, and cancer (Cybulsky, 2017).

Moreover, there is a complex interplay between ER stress and inflammation. ER stress can activate inflammatory signaling pathways, such as the NF-κB pathway, through various mechanisms. For example, IRE1α can recruit the adaptor protein tumor necrosis factor receptor-associated factor 2 (TRAF2) and activate the IκB kinase (IKK) complex, leading to NF-κB activation (Zhang and Kaufman, 2008). Additionally, PERK-mediated phosphorylation of eukaryotic translation initiation factor 2α (eIF2α) can lead to the preferential translation of activating transcription factor 4 (ATF4), which can induce the expression of pro-inflammatory cytokines (Zhang and Kaufman, 2008). Conversely, inflammation can also exacerbate ER stress, creating a vicious cycle that contributes to the progression of various diseases (Zhang and Kaufman, 2008; Cybulsky, 2017).

Given the critical role of ER stress in cellular homeostasis and disease pathogenesis, understanding the regulatory mechanisms that modulate the UPR and its crosstalk with inflammatory signaling pathways is of great importance. In this context, SIRT1 has emerged as a key player in the regulation of ER stress and inflammation (Prola et al., 2017). However, the precise mechanisms by which SIRT1 exerts its protective effects and the potential therapeutic implications of targeting SIRT1 in ER stress-related diseases remain to be fully elucidated.

**FIGURE 1:**
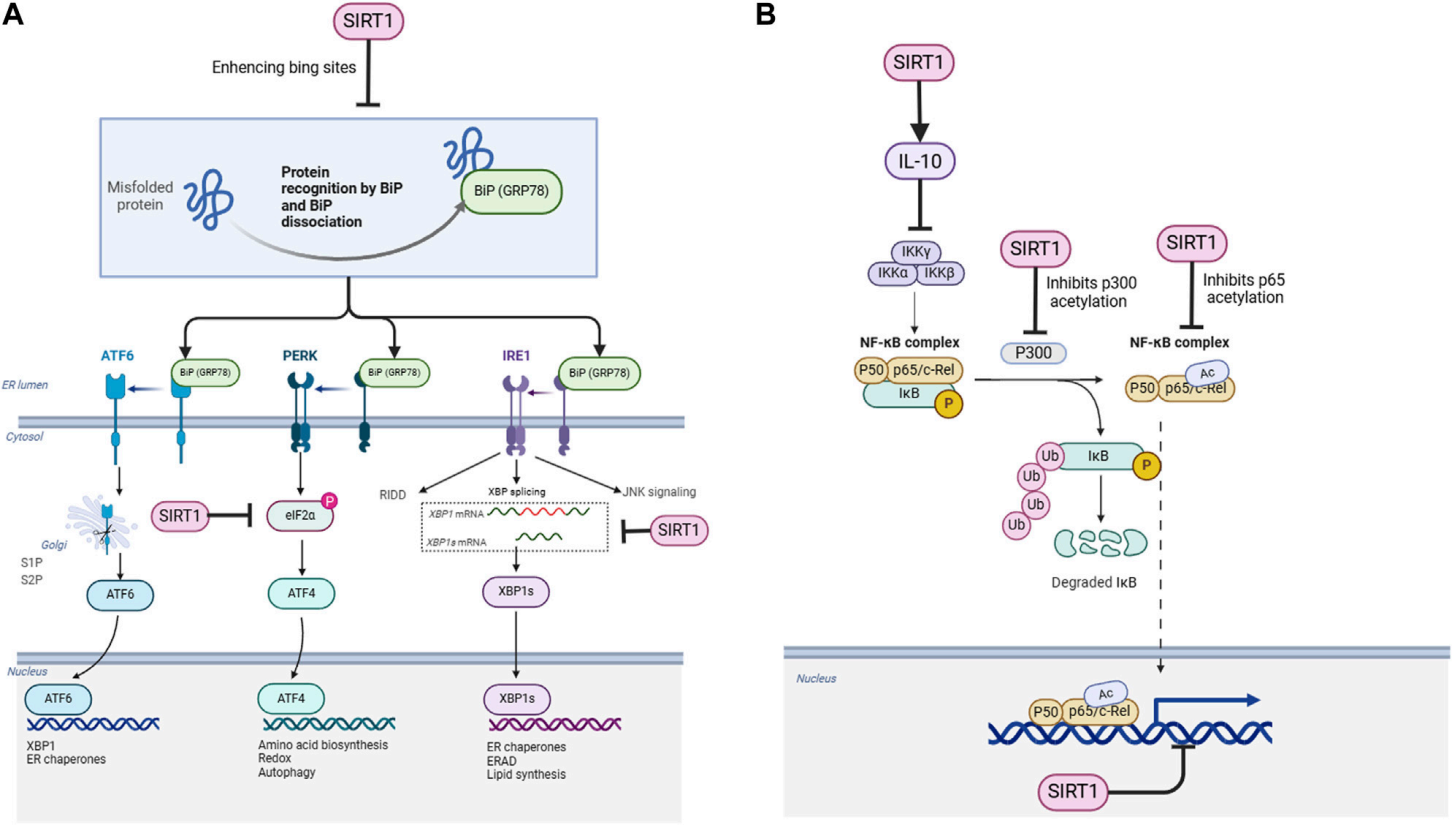
Schematic representation of the regulatory mechanisms of SIRT1 on ER stress and NF-κB signaling pathways. **(A)** SIRT1 enhances the binding of GRP78 (BiP) to misfolded proteins, facilitating their recognition and dissociation from ER stress sensors (PERK, IRE1, and ATF6). SIRT1 also deacetylates eIF2α, IRE1, and XBP1s, modulating their activities in the UPR pathways. **(B)** SIRT1 deacetylates the NF-κB p65 subunit (RelA) directly and indirectly through p300, inhibiting NF-κB transcriptional activity. SIRT1 also activates AMPK, which inhibits IKK and NF-κB signaling. Additionally, SIRT1 upregulates the expression of the anti-inflammatory cytokine IL-10, forming a negative feedback loop to suppress NF-κB activity.

As a key transcription factor involved in innate and adaptive immunity, NF-κB plays a central role in inflammatory responses, metabolic regulation, and cell proliferation. Persistent abnormal activation of NF-κB can cause chronic inflammation and tissue damage, which is a critical step in the development of many diseases (Lawrence, 2009). Therefore, inhibiting excessive ER stress and NF-κB activation may provide new entry points for the prevention and treatment of related diseases. Interestingly, SIRT1 has been shown to exert significant protective effects in multiple disease models by negatively regulating ER stress and the NF-κB pathway (Figure 1) (Prola et al., 2017). However, the enthusiasm surrounding these findings must be tempered by the recognition that the biological systems involved are highly complex, and the potential for unintended consequences cannot be overlooked. This review will focus on the recent advances in the molecular mechanisms and biological significance of SIRT1 in regulating ER stress and the NF-κB pathway, while also critically examining the limitations and uncertainties in the current knowledge.

## 2 Regulatory mechanisms of SIRT1 on ER stress

### 2.1 SIRT1 deacetylates GRP78 to enhance its binding to ER stress sensors

Glucose-regulated protein 78 (GRP78) is a classic marker of ER stress and an important ER chaperone protein. Under non-stress conditions, GRP78 binds to the three ER stress sensors, namely, PKR-like ER kinase (PERK), inositol-requiring enzyme 1α (IRE1α), and activating transcription factor 6 (ATF6), on the ER lumen side, keeping them in an inactive state. When ER stress occurs, the accumulated unfolded proteins competitively bind to GRP78, leading to its dissociation from the stress sensors, thereby activating the UPR (Ibrahim et al., 2019). Studies have shown that in hepatocytes and cardiomyocytes, SIRT1 can bind to and directly deacetylate GRP78, thereby enhancing its affinity for stress sensors and inhibiting the activation of ER stress sensors (Wang et al., 2011). When SIRT1 is knocked down, ER stress is aggravated in the liver of diabetic mice, accompanied by increased acetylation levels of GRP78. These findings suggest that SIRT1 can inhibit the excessive activation of the three UPR pathways by deacetylating GRP78, thereby alleviating ER stress. However, the precise mechanisms by which SIRT1-mediated deacetylation of GRP78 enhances its binding to ER stress sensors remain to be fully elucidated.

### 2.2 SIRT1 deacetylates IRE1α to inhibit XBP1 splicing

As one of the three major ER stress sensors, IRE1α possesses both serine/threonine kinase and endoribonuclease activities. During ER stress, GRP78 dissociates from IRE1α, which then undergoes oligomerization and autophosphorylation. Its endoribonuclease domain is activated and selectively splices X-box binding protein 1 (XBP1) mRNA, producing transcriptionally active XBP1s, which in turn initiates the expression of downstream UPR-related genes (Calfon et al., 2002). Research has revealed that SIRT1 can directly bind to and specifically deacetylate IRE1α at lysine 599 (K599) (Yao et al., 2010). This residue is located in the RNase domain of IRE1α, and its acetylation modification can enhance the endoribonuclease activity of IRE1α. Therefore, SIRT1 inhibits the splicing and activation of XBP1 by removing the acetyl group at K599, thereby attenuating the excessive activation of the IRE1α-XBP1 signaling pathway. Knockdown of SIRT1 can lead to increased acetylation levels of IRE1α and enhanced XBP1 splicing (Yao et al., 2010). This suggests that SIRT1 can selectively inhibit the IRE1α-XBP1 branch by deacetylating IRE1α, finely regulating the ER stress response at the IRE1α level. However, the biological significance of this selective regulation and its potential implications for disease pathogenesis remain to be clarified.

### 2.3 SIRT1 deacetylates eIF2α to inhibit protein synthesis

PERK is another important ER stress sensor that can undergo oligomerization and autophosphorylation during stress and specifically phosphorylate its downstream substrate, eukaryotic translation initiation factor 2α (eIF2α), leading to a general reduction in protein synthesis to alleviate the protein load on the ER (Harding et al., 2000). Studies have revealed that SIRT1 can mediate the deacetylation of eIF2α, thereby affecting the PERK-eIF2α signaling pathway (Wang et al., 2012). The acetylation modification of eIF2α can promote its phosphorylation and enhance PERK signaling, while SIRT1 can inhibit its phosphorylation level by removing the acetyl groups from eIF2α, antagonizing the suppression of protein synthesis to a certain extent, thereby alleviating ER stress (Wang et al., 2012). However, the functional consequences of this regulation and its potential impact on cellular homeostasis and disease progression require further investigation.

### 2.4 SIRT1 deacetylates XBP1 to enhance its transcriptional activity

In addition to the IRE1α-XBP1 pathway, ATF6 can also be activated during ER stress, initiating the transcription of downstream molecular chaperones and ER-associated degradation (ERAD) components. Research has shown that SIRT1 can bind to and directly deacetylate spliced XBP1 (XBP1s), thereby enhancing the transcriptional activity of XBP1s (Koga et al., 2015). Overexpression of SIRT1 can upregulate multiple target genes of XBP1s, including molecular chaperones such as GRP78 and protein disulfide isomerase (PDI), as well as ERAD components such as ER degradation-enhancing α-mannosidase-like protein (EDEM) and Derlin-2 (Koga et al., 2015). These gene products can improve the folding capacity of the ER and accelerate the clearance of unfolded proteins, thereby alleviating ER stress. Thus, SIRT1 exerts another pathway to alleviate ER stress by deacetylating XBP1s. However, the physiological relevance of this regulation and its potential impact on cellular adaptation to ER stress remain to be elucidated.

In summary, SIRT1 can synergistically inhibit ER stress at different levels and through multiple mechanisms by deacetylating key molecules in the ER stress pathway, such as GRP78, IRE1α, eIF2α, and XBP1s (Table 1). Decreased expression or activity of SIRT1 can weaken its protective effects against ER stress, leading to excessive activation of the UPR and increased cell apoptosis. Therefore, SIRT1 activators may exert therapeutic effects in various disease states by inhibiting abnormal ER stress. However, the complex nature of the ER stress response and the potential for compensatory mechanisms necessitate a cautious approach in targeting SIRT1 for therapeutic intervention.

**TABLE 1:** SIRT1-mediated deacetylation of key molecules in the ER stress pathway and their biological effects.

ER stress molecule	SIRT1 deacetylation site	Biological effects	References
GRP78	To be determined	Enhances GRP78 binding to ER stress sensors, inhibits UPR	Wang et al. (2011)
IRE1α	K599	Inhibits IRE1α endoribonuclease activity, suppresses XBP1 splicing	Yao et al. (2010)
eIF2α	To be determined	Inhibits eIF2α phosphorylation, antagonizes protein synthesis suppression	Wang et al. (2012)
XBP1s	To be determined	Enhances XBP1s transcriptional activity, upregulates molecular chaperones and ERAD components	Koga et al. (2015)

## 3 Inhibitory effects of SIRT1 on the NF-κB inflammatory pathway

### 3.1 SIRT1 directly deacetylates the NF-κB p65 subunit to inhibit its transcriptional activity

In addition to regulating ER stress, SIRT1 can also directly inhibit the NF-κB inflammatory signaling pathway through various mechanisms. Studies have first discovered that SIRT1 can directly bind to the NF-κB p65 subunit and specifically remove the acetylation modification at lysine 310 (K310), thereby inhibiting the transcriptional activity of p65 (Yeung et al., 2004). The acetylation of p65 at K310 can enhance its DNA binding ability, which is essential for the transcriptional activation of NF-κB (Chen et al., 2002). SIRT1 gene knockout cells and SIRT1 inhibitor treatment can both lead to increased acetylation levels of p65 and enhanced expression of NF-κB target genes, while SIRT1 overexpression or SIRT1 activators can inhibit the transcriptional activity of NF-κB (Yeung et al., 2004). Furthermore, in vascular endothelial cells, SIRT1 can inhibit the acetylation of p65 and the expression of adhesion molecules such as intercellular adhesion molecule-1 (ICAM-1) and vascular cell adhesion molecule-1 (VCAM-1) induced by tumor necrosis factor α (TNFα) (Lee et al., 2008). These findings indicate that the deacetylation of p65 is an important mechanism by which SIRT1 inhibits the NF-κB signaling pathway. However, the biological significance of this regulation in the context of specific inflammatory diseases requires further clarification.

### 3.2 SIRT1 indirectly inhibits p65 acetylation by deacetylating p300

In addition to directly removing the acetylation modification of p65, SIRT1 can also indirectly inhibit NF-κB activity by deacetylating p300 (Liu et al., 2012). p300 is a histone acetyltransferase that can catalyze histone acetylation and initiate gene transcription, and it is also an acetyltransferase for NF-κB p65. Interestingly, the acetyltransferase activity of p300 itself is also regulated by acetylation modification, and the acetylation of specific lysine residues can enhance the enzymatic activity of p300 (Thompson et al., 2004). Studies have found that SIRT1 can directly bind to and deacetylate p300, thereby inhibiting the acetyltransferase activity of p300 and reducing the acetylation modification of p65 (Liu et al., 2012). In the brain and liver tissues of mice treated with SIRT1 inhibitors, increased acetylation levels of p300 and p65 and enhanced expression of NF-κB inflammatory genes can be observed (Liu et al., 2012). This reveals the cascading regulatory mechanism by which SIRT1 indirectly attenuates p65 acetylation by deacetylating p300. However, the physiological relevance of this indirect regulation and its potential implications for inflammatory responses *in vivo* remain to be elucidated.

### 3.3 SIRT1 inhibits the IKKβ/NF-κB inflammatory signaling by activating AMPK

In addition to directly or indirectly removing the acetylation modification of NF-κB p65, SIRT1 can also inhibit the upstream kinase of NF-κB, IκB kinase β (IKKβ), by activating AMP-activated protein kinase (AMPK). AMPK is a key regulator of cellular energy metabolism, and its activity can be enhanced by SIRT1-mediated deacetylation (Cantó et al., 2009). Activated AMPK can inhibit the activity of IKKβ, reduce the degradation of IκBα, and thereby inhibit the nuclear translocation of p65 and NF-κB-mediated transcription (Bess et al., 2011). In the liver and adipose tissues of obese mice, decreased SIRT1 expression is accompanied by suppressed AMPK activity and activated NF-κB signaling pathway (Yoshizaki et al., 2009). Administration of the SIRT1 activator resveratrol can restore the phosphorylation level of AMPK, inhibit the IKKβ/NF-κB pathway, and thus improve insulin resistance (Yoshizaki et al., 2009). These findings suggest that the activation of the SIRT1/AMPK pathway can inhibit the inflammatory signaling of NF-κB at the upstream level and exert anti-inflammatory protective effects. However, the precise mechanisms by which SIRT1 activates AMPK and the potential for tissue-specific regulation remain to be fully elucidated.

### 3.4 SIRT1 negatively regulates the NF-κB inflammatory response by upregulating IL-10

In addition to directly or indirectly inhibiting NF-κB activity, SIRT1 can also exert anti-inflammatory effects by inducing the expression of the anti-inflammatory cytokine interleukin-10 (IL-10). Studies have found that in macrophages, SIRT1 can be recruited to the promoter region of IL-10 and upregulate IL-10 transcription by deacetylating histone H3K9 and H4K16 (Zhang et al., 2020). IL-10 can inhibit the NF-κB inflammatory signaling pathway, forming a negative feedback regulation. Overexpression of SIRT1 can significantly increase IL-10 secretion in lipopolysaccharide (LPS)-stimulated macrophages and reduce the production of inflammatory cytokines such as TNFα and interleukin-6 (IL-6), while knockout of SIRT1 leads to decreased IL-10 levels and exacerbated inflammatory responses (Zhang et al., 2020). Furthermore, in a mouse model of sepsis, the SIRT1 activator resveratrol can alleviate inflammatory responses and organ damage by upregulating IL-10 expression, suggesting that SIRT1-induced IL-10 can significantly antagonize the inflammatorycascade amplification mediated by NF-κB (Li et al., 2013). However, the biological significance of this negative feedback regulation in the context of specific inflammatory diseases and the potential for therapeutic targeting of the SIRT1-IL-10 axis remain to be explored.

In summary, SIRT1 can negatively regulate the NF-κB inflammatory signaling pathway through various mechanisms, including directly removing the acetylation modification of the p65 subunit, indirectly inhibiting p65 acetylation by deacetylating p300, activating the AMPK pathway to inhibit the upstream kinase IKKβ of NF-κB, and negatively regulating NF-κB activity by upregulating the anti-inflammatory cytokine IL-10 (Table 2). The inhibitory effects of SIRT1 on the NF-κB signaling pathway can significantly alleviate chronic inflammatory responses in tissues and exert important protective effects in metabolic diseases such as insulin resistance and atherosclerosis, as well as in inflammation-related diseases. However, the complex nature of inflammatory responses and the potential for compensatory mechanisms highlight the need for further research to fully elucidate the therapeutic potential of targeting SIRT1 in these contexts.

**TABLE 2:** SIRT1-mediated regulatory mechanisms of the NF-κB inflammatory signaling pathway.

Regulatory mechanism	SIRT1 target	Biological effects	References
Direct deacetylation	NF-κB p65 subunit K310	Inhibits p65 transcriptional activity	Chen et al. (2002), Yeung et al. (2004)
Indirect deacetylation	p300	Inhibits p300 activity, thereby reducing p65 acetylation	Thompson et al. (2004), Liu et al. (2012)
Activation of AMPK pathway	AMPK	Inhibits IKKβ activity, suppresses NF-κB activation	Cantó et al. (2009), Yoshizaki et al. (2009), Bess et al. (2011)
Induction of IL-10 expression	Histone H3K9 and H4K16	Upregulates anti-inflammatory cytokine IL-10, negative feedback inhibition of NF-κB	Li et al. (2013), Zhang et al. (2020)

## 4 Biological significance of SIRT1 regulation on ER stress and the NF-κB pathway

### 4.1 SIRT1 improves metabolic diseases by inhibiting ER stress

The occurrence and development of metabolic diseases such as obesity and type 2 diabetes are closely related to ER stress. Chronic ER stress is widespread in metabolic tissues such as adipocytes, hepatocytes, and pancreatic β cells, leading to increased insulin resistance, inflammatory responses, and cell apoptosis (Hotamisligil, 2010). Studies have shown that in obesity and type 2 diabetes models, SIRT1 expression and activity are significantly decreased, accompanied by a significant increase in ER stress (Liu et al., 2014). Overexpression of SIRT1 or administration of SIRT1 activators can improve insulin sensitivity and glucose homeostasis, reduce inflammatory responses in adipose tissue and liver, and exert metabolic protective effects by inhibiting ER stress (Li et al., 2011). These studies suggest that SIRT1 can play an important therapeutic role in metabolic diseases such as obesity and type 2 diabetes by suppressing chronic ER stress. However, the long-term efficacy and safety of targeting SIRT1 in these diseases require further evaluation in clinical studies.

### 4.2 SIRT1 alleviates myocardial ischemia-reperfusion injury by inhibiting ER stress

Myocardial ischemia-reperfusion injury is an exacerbation of tissue damage that occurs when blood flow is restored after myocardial infarction, and its pathological mechanisms involve multiple factors such as oxidative stress, calcium overload, and ER stress (Groenendyk et al., 2013). Studies have found that ER stress is significantly activated during myocardial ischemia-reperfusion, causing cardiomyocyte apoptosis and aggravating myocardial dysfunction (Thuerauf et al., 2006). Activation of SIRT1 can alleviate myocardial ischemia-reperfusion injury by inhibiting ER stress-dependent apoptotic pathways (Guo et al., 2015). Furthermore, SIRT1 can also promote ER calcium homeostasis by upregulating key molecules such as sarco/endoplasmic reticulum Ca2+-ATPase (SERCA), thereby exerting cardioprotective effects in myocardial ischemia-reperfusion injury (Zhang et al., 2017). However, the translational potential of these findings and the optimal strategies for targeting SIRT1 in the context of myocardial ischemia-reperfusion injury remain to be determined.

### 4.3 SIRT1 alleviates neurodegenerative pathology by inhibiting ER stress and the NF-κB pathway

The pathogenesis of neurodegenerative diseases such as Alzheimer’s disease and Parkinson’s disease is complex, involving multiple pathological processes such as neuronal protein misfolding, mitochondrial dysfunction, and chronic inflammation, with ER stress and NF-κB inflammatory responses playing key roles (Huang and Mucke, 2012). Studies have shown that in the brain tissues of Alzheimer’s disease patients and animal models, SIRT1 expression and activity are significantly reduced, accompanied by pathological changes such as β-amyloid (Aβ) aggregation, tau protein hyperphosphorylation, and exacerbated neuroinflammation (Julien et al., 2009). Overexpression of SIRT1 or administration of SIRT1 activators can alleviate Aβ aggregation and tau protein phosphorylation and exert significant neuroprotective effects by inhibiting ER stress and the NF-κB inflammatory pathway (Min et al., 2010). Similarly, in Parkinson’s disease animal models, SIRT1 can also improve the survival of dopaminergic neurons and motor function by inhibiting ER stress and neuroinflammatory responses induced by α-synuclein aggregation (Donmez et al., 2012). These studies demonstrate that SIRT1 exerts neuroprotective effects in various neurodegenerative diseases by coordinately regulating ER stress and the NF-κB pathway. However, the complex etiology and progressive nature of neurodegenerative diseases pose significant challenges for the development of effective therapies targeting SIRT1.

### 4.4 SIRT1 alleviates inflammatory diseases by inhibiting ER stress and the NF-κB pathway

Inflammatory reaction and tissue damage are the issue of many chronic diseases with ER stress and NF-κB pathway being the ones that are primarily responsible for the exacerbation of the inflammatory chain reaction. Research has determined that SIRT1 expression and activity are down in patients with chronic inflammatory diseases such as rheumatoid arthritis and inflammatory bowel disease, which are associated with the long-term activation of ER stress and the NF-kB signaling pathway (Zhang et al., 2009). In rheumatoid arthritis animal models, SIRT1 overexpression or administration of SIRT1 activators can markedly suppress the ER stress and the NF-κB activation of synovial cell, which basically decreased synovial inflammation and cartilage destruction (Niederer et al., 2011). In addition, in the experimental setting of animal models of inflammatory bowel disease, SIRT1 can also reduce intestinal inflammatory responses and tissue damage by inhibiting ER stress response and the activity of the NF-κB pathway in the intestinal epithelial and immune cells, which maintain the intestinal barrier function (Caruso et al., 2014). These observations point that a more precise intervention of SIRT1-ER stress-NF-κB pathway may be a novel cellular mechanism for the treatment of inflammation-associated diseases (Table 3). The application of the identified health benefits needed to be further studied especially on the possibility of side-effects and suitable patient population.

**TABLE 3:** Protective effects of SIRT1 in diseases and their mechanisms.

Disease type	SIRT1 protective effects	Potential mechanisms	References
Metabolic diseases (e.g., obesity, type 2 diabetes)	Improves insulin resistance, reduces inflammation	Inhibits ER stress, improves metabolic homeostasis	Hotamisligil (2010), Li et al. (2011), Liu et al. (2014)
Myocardial ischemia-reperfusion injury	Reduces cardiomyocyte apoptosis, improves cardiac function	Inhibits ER stress-dependent apoptotic pathways	Thuerauf et al. (2006), Groenendyk et al. (2013), Guo et al. (2015), Zhang et al. (2017)
Neurodegenerative diseases (e.g., Alzheimer’s disease, Parkinson’s disease)	Reduces protein aggregation and neuroinflammation, provides neuroprotection	Inhibits ER stress and NF-κB pathway	Julien et al. (2009), Min et al. (2010), Donmez et al. (2012), Huang and Mucke (2012)
Inflammatory diseases (e.g., rheumatoid arthritis, inflammatory bowel disease)	Reduces tissue inflammation and damage	Inhibits ER stress and NF-κB pathway	Zhang et al. (2009), Niederer et al. (2011), Caruso et al. (2014)

### 4.5 Crosstalk between ER stress and NF-κB signaling during regulation by SIRT1 in various diseases

The interplay between ER stress and inflammation, particularly NF-κB signaling, has been increasingly recognized as a critical factor in the pathogenesis of various diseases (Zhang and Kaufman, 2008; Cybulsky, 2017). SIRT1, as a key regulator of both ER stress and NF-κB pathways, may play a pivotal role in modulating this crosstalk. While the precise mechanisms remain to be fully elucidated, several lines of evidence suggest that SIRT1 may attenuate the vicious cycle between ER stress and NF-κB activation.

Firstly, SIRT1-mediated deacetylation of the NF-κB p65 subunit can directly suppress its transcriptional activity (Chen et al., 2002; Yeung et al., 2004), which may reduce the expression of pro-inflammatory genes and alleviate the protein folding burden on the ER. This, in turn, could attenuate ER stress and the associated UPR activation. Secondly, SIRT1 has been shown to deacetylate key molecules in the UPR pathways, such as IRE1α and XBP1s (Yao et al., 2010; Koga et al., 2015), which may inhibit their ability to activate NF-κB signaling. For example, deacetylation of IRE1α by SIRT1 may disrupt its interaction with TRAF2 and subsequent activation of IKK and NF-κB (Zhang and Kaufman, 2008).

Furthermore, SIRT1-mediated activation of AMPK has been implicated in suppressing both ER stress and NF-κB signaling (Cantó et al., 2009; Bess et al., 2011). AMPK can phosphorylate and inhibit the IKKβ subunit, thereby attenuating NF-κB activation (Bess et al., 2011). Additionally, AMPK activation has been shown to alleviate ER stress by promoting autophagy and reducing protein synthesis (Kim et al., 2011). Thus, SIRT1-AMPK signaling may represent another avenue through which SIRT1 can modulate the crosstalk between ER stress and NF-κB pathways.

In conclusion, while the exact mechanisms remain to be fully characterized, emerging evidence suggests that SIRT1 may play a crucial role in attenuating the vicious cycle between ER stress and NF-κB signaling. This highlights the potential of targeting SIRT1 as a therapeutic strategy for diseases characterized by chronic ER stress and inflammation. However, further research is needed to elucidate the complex interplay between these pathways and to develop targeted interventions that exploit the protective effects of SIRT1.

## 5 Summary and outlook

As an NAD + -dependent histone deacetylase, SIRT1 plays a key role in the regulation of various stress responses and signaling pathways through epigenetic modifications, and it is involved in multiple biological processes such as metabolism and inflammation. This review summarizes the research progress on the inhibitory effects of SIRT1 on ER stress by deacetylating key molecules in the ER stress pathway, such as GRP78, IRE1α, eIF2α, and XBP1s, as well as the negative regulation of NF-κB inflammatory signaling by SIRT1 through mechanisms such as removing the acetylation of the NF-κB p65 subunit, inhibiting p300 activity, activating the AMPK pathway, and inducing IL-10 expression. These studies reveal the important biological significance of SIRT1 in coordinately regulating ER stress and the NF-κB pathway in various pathological processes such as metabolic diseases, ischemia-reperfusion injury, neurodegenerative diseases, and inflammatory diseases. However, it is important to recognize that the complexity of the biological systems involved and the potential for unintended consequences necessitate a cautious approach in targeting SIRT1 for therapeutic intervention.

Although significant progress has been made in the research on SIRT1 regulation of ER stress and the NF-κB pathway, further in-depth understanding of its mechanisms of action and clinical application prospects is still needed. Future research should be conducted in the following aspects: First, further elucidate the precise molecular mechanisms of SIRT1 regulation of ER stress and the NF-κB pathway in specific cells and tissues, and identify new SIRT1 deacetylation substrates and interacting proteins. Second, use tissue-specific SIRT1 knockout and overexpression animal models to deeply explore the role of SIRT1 regulation of ER stress and inflammatory responses in the development of specific diseases. Third, screen for highly efficient and specific SIRT1 activators and evaluate their therapeutic effects and safety in animal models of ER stress and inflammation-related diseases, laying the foundation for their clinical application. Fourth, design prospective clinical studies to explore the expression profile and activity changes of SIRT1 and its related targets in the ER stress and inflammatory signaling pathways in related diseases, and identify new diagnostic and prognostic markers. Fifth, conduct drug development and clinical trials based on the SIRT1-ER stress-NF-κB pathway to promote its application in the prevention and treatment of metabolic diseases, cardiovascular and cerebrovascular diseases, neurodegenerative diseases, and inflammatory diseases.

In conclusion, SIRT1 plays a crucial role in maintaining body homeostasis and the development of various diseases through the fine-tuned regulation of ER stress and the NF-κB pathway. In-depth elucidation of the mechanisms of action and biological functions of SIRT1 is of great significance for expanding our understanding of the pathogenesis of related diseases and exploring novel preventive and therapeutic strategies targeting SIRT1. However, the translation of these findings into clinical applications requires a rigorous and holistic approach, taking into account the complex nature of the biological systems involved and the potential for adverse effects. Future research requires multidisciplinary collaboration to deeply explore the patterns and significance of SIRT1 regulation of ER stress and inflammatory responses at multiple levels, including molecular mechanisms, animal models, and clinical translation, ultimately realizing the transformation from basic research to clinical application and benefiting human health. While the challenges are significant, the potential rewards are great, and the continued pursuit of this line of research holds great promise for the development of new and effective therapies for a wide range of diseases.
